# On-chip manufacturing of synthetic proteins for point-of-care therapeutics

**DOI:** 10.1038/s41378-019-0051-8

**Published:** 2019-03-25

**Authors:** Travis W. Murphy, Jiayuan Sheng, Lynette B. Naler, Xueyang Feng, Chang Lu

**Affiliations:** 1 Department of Chemical Engineering, Virginia Tech, Blacksburg, VA 24061 USA; 2 Department of Biological Systems Engineering, Virginia Tech, Blacksburg, VA 24061 USA

**Keywords:** Engineering, Chemistry

## Abstract

Therapeutic proteins have recently received increasing attention because of their clinical potential. Currently, most therapeutic proteins are produced on a large scale using various cell culture systems. However, storing and transporting these therapeutic proteins at low temperatures makes their distribution expensive and problematic, especially for applications in remote locations. To this end, an emerging solution is to use point-of-care technologies that enable immediate and accessible protein production at or near the patient’s bedside. Here we present the development of “Therapeutics-On-a-Chip (TOC)”, an integrated microfluidic platform that enables point-of-care synthesis and purification of therapeutic proteins. We used fresh and lyophilized materials for cell-free synthesis of therapeutic proteins on microfluidic chips and applied immunoprecipitation for highly efficient, on-chip protein purification. We first demonstrated this approach by expressing and purifying a reporter protein, green fluorescent protein. Next, we used TOC to produce cecropin B, an antimicrobial peptide that is widely used to control biofilm-associated diseases. We successfully synthesized and purified cecropin B at 63 ng/μl within 6 h with a 92% purity, followed by confirming its antimicrobial functionality using a growth inhibition assay. Our TOC technology provides a new platform for point-of-care production of therapeutic proteins at a clinically relevant quantity.

## Microfluidics: Providing therapeutic proteins at the point of care

A microfluidic reactor enables researchers to synthesize therapeutic proteins at the point of care. Therapeutic proteins are usually produced in a central location, requiring costly refrigeration and transport to the location where they’re administered. This hinders care for patients in remote or resource-scarce areas. Engineers from the United States’ Virginia Tech, led by Chang Lu, have now developed a microfluidic reactor that uses cell components and a DNA template, completed by a protein purification device. The team validated their device by producing an easily quantifiable protein GFP, before producing therapeutically viable concentrations of cecropin B, a protein used in the treatment of microbial infections. The functionality of the synthesized cecropin B was confirmed by its successful inhibition of *Escherichia coli*. The devices are low-cost, and future research could extend their utility to other clinically beneficial proteins.

## Introduction

The ability to synthesize proteins in a point-of-care setting is an important step for reducing costs associated with storage and transportation of therapeutic proteins and enabling their widespread application in resource-poor regions^1^. Therapeutic proteins have a wide range of functions, including replacing or supplementing existing proteins or pathways, providing novel activity, inhibiting microorganisms, and delivering drugs or other proteins^2^. Currently, the majority of therapeutic proteins are produced on a large scale using various cell culture systems, including recombinant *Escherichia coli*^3^ or yeast^4^, mammalian^5^, and plant^6^ cells. These proteins are then globally distributed from centralized foundries^7^. These therapeutic proteins have a limited shelf life and require shipment and storage at low temperatures^8^. The ability to produce therapeutic proteins on the spot, removing the need for cold storage, will greatly benefit remote regions and patients in low-resource settings.

There have been efforts in developing point-of-use therapeutic protein synthesis. The current state of the art is a refrigerator-sized system that requires 2 d for production and purification, producing ~ 800 doses or more per day^9^. Unfortunately, the capital cost of such a system will make it an unlikely solution for the developing world where the need is more for speedy production of different reagents for different diseases over producing mass quantities that require storage.

**Fig. 1:**
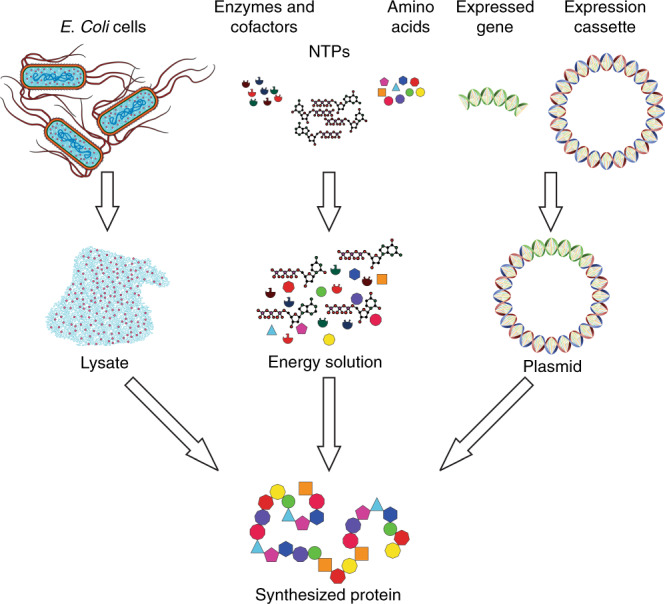
Overview of cell-free protein synthesis system. The synthesis system is made of three components: lysate, energy solution, and plasmid DNA. The lysate is collected from *E. coli* cells by cell lysis and prepared using ultracentrifugation. The energy solution is composed of nucleoside triphosphates (NTPs), amino acids, enzymes, and cofactors. The plasmid DNA is made by inserting the expressed gene of interest into an expression cassette. The three components are combined and incubated to synthesize protein of interest

In this work, we developed a highly integrated TOC system that enabled rapid and point-of-care synthesis and purification of therapeutic proteins based on a cell-free protein synthesis (CFPS) process. The CFPS system expresses recombinant proteins in vitro without the use of living cells (Fig. 1)^10–12^. As an alternative to cell-based protein synthesis, CFPS is particularly suited for point-of-care production due to the convenience in storage of starting materials, which can be lyophilized and remain stable in a broad temperature range^13–15^. We examined the production of two protein targets in this work, green fluorescent protein (GFP) and cecropin B. Cecropin B is an antimicrobial protein, which shows proven results for treating fungal infections^16^ and potential for treating cancer^17^. Cecropin B has been found to have a minimum inhibitory concentration (MIC) of 9.5 ng/μl^16^. We demonstrated a microfluidic system that combined protein synthesis and purification and was capable of producing an antimicrobial peptide, cecropin B, at a clinically relevant dose (up to 63 ng/μl). The integrated microfluidic system utilized a novel tubing storage reservoir to interface a continuous-flow production section and a batch protein purification section.

## Results

We sought to develop and validate the proposed cell-free protein synthesis and purification (CFPS + P) microfluidic system in three developmental phases: CFPS reactor design, purification reactor design, and integrated CFPS + P system design. All of our devices were fabricated using soft lithography-based polydimethylsiloxane (PDMS) molding. For our devices using micromechanical valves, we utilized multilayer fabrication techniques^18^, which are further explained in the methods section.

**Fig. 2:**
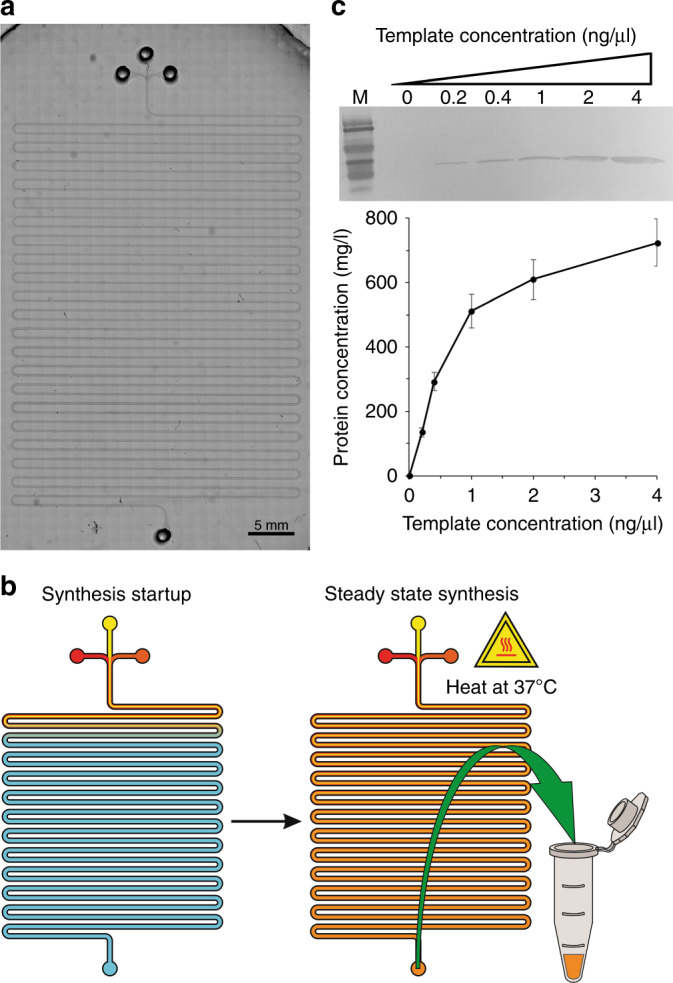
Protein synthesis in a serpentine channel. **a** A microscopic image of the synthesis module. **b** The steps for synthesis: start of synthesis and steady state synthesis. **c** Western blotting of GFP produced in the CFPS system and the protein yield with different concentrations of the plasmid template. Mouse 6 × His tag monoclonal primary antibody and HRP-conjugated goat anti-mouse IgG (H + L) secondary antibody were used to detect the target protein. Concentrations listed are the concentrations in the final reaction volume

In the first phase of CFPS reactor design, we fabricated a serpentine-channel microfluidic chip for the CFPS system (Fig. 2a). This device was similar to one used in published studies^19–24^. The microfluidic device had inlets connected to a syringe pump and placed on the heating stage of a microscope (Supplementary Figure S1a). The microfluidic device had three inlets, which fed cell lysate, CFPS reaction buffer (energy solution), and DNA template (plasmid) into an ~130 cm long serpentine channel with one outlet. With a width of 200 μm and a depth of 50 μm, the serpentine microfluidic channel had a volume of ~13.2 μl. The three reaction components were fed at a combined flow rate of 0.15 μl/min (0.05 μl/min per reagent) driven by a syringe pump, giving a residence time of 1.5 h. The reactor was heated using a stage heater, allowing the reaction to take place at 37 °C. The channel reaches 37 °C within 1 min of heating as shown in COMSOL modeling (Supplementary Figure S2). This allowed for on-chip diffusion-based mixing and reaction (Fig. 2b).

We validated the device operation by synthesizing GFP, as it is a simple and easily quantifiable target for analysis. We tried various concentrations of the DNA template to explore the effect on the final protein concentration, as shown in Fig. 2c. In general, for the range of template concentration tested (0–4 ng/µl), the produced protein concentration continued to increase with the higher template concentration. Using a template concentration of 4 ng/µl, the device was able to produce 50 μl GFP (4 reactor volumes) at a concentration of 700 ng/μl in 7.5 h (i.e., 35 μg protein). Our system utilized a continuous-flow system, which allowed fine tuning of the amount of protein synthesized with a constant reaction time of 1.5 h. In comparison, previous microfluidic systems required over 2.5 h to perform protein synthesis^19^ and off-chip methods used up to 8 h reaction time and were carried out in a batch system^25^.

**Fig. 3:**
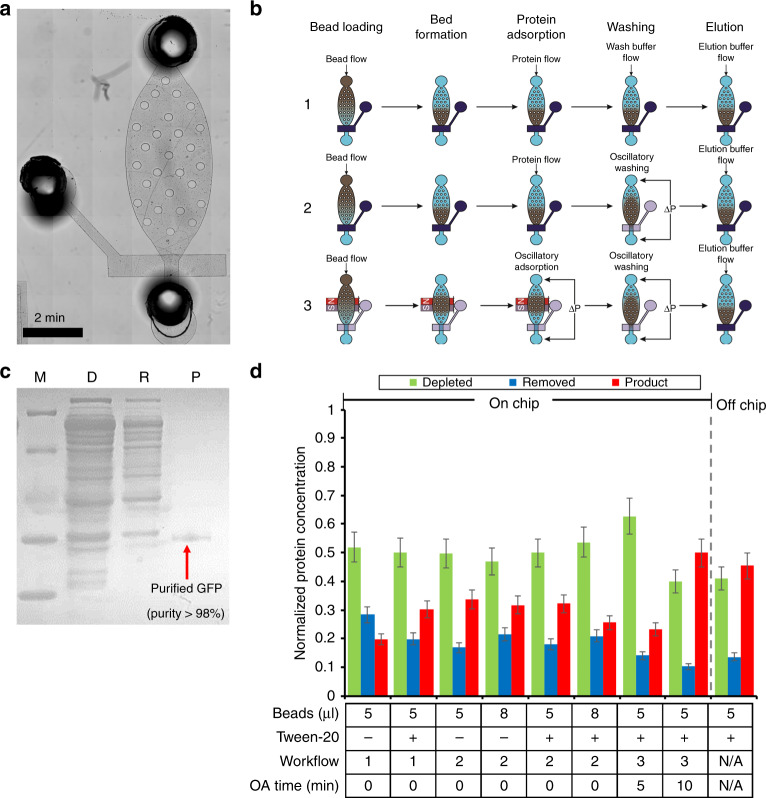
Protein purification in a microfluidic chamber. **a** A microscopic image of the purification module. **b** Overview of the purification procedure (including bead loading, bed formation, protein adsorption, washing, and elution) by three different workflows. Workflow 1 uses flow adsorption and washing steps. Workflow 2 uses flow adsorption and oscillatory washing. Workflow 3 uses oscillatory adsorption and washing. Dark blue denotes a closed valve, where transparency denotes an open valve. **c** SDS-PAGE of GFP purified by the purification chip. M (Marker); D (Depleted): CFPS reaction mix after bead absorption; R (Removed): removed contaminates in the purification buffer; P (Product): purified GFP in the elution buffer. **d** The purification step optimization. Optimization was conducted by examining 4 conditions. (1) Ni-NTA bead volume of 5 or 8 µl; (2) 0.5% Tween-20 added into purification and elution buffers. (3) Different workflows. (4) Different oscillatory adsorption times

In the second phase, we designed a microfluidic device for protein purification based on our previous work in high efficiency adsorption and washing (Fig. 3a)^26,27^. Such technology has not been applied to protein purification previously. The two-layer microfluidic device was composed of 30 μm deep control channels for pneumatic valve actuation, bound to the glass-slide substrate, and 50 μm deep fluidic channels for sample transport and manipulation bound on top, separated by a thin PDMS membrane. The device was operated using pneumatic actuation of solenoid valves to control the single micromechanical valve (Supplementary Figure S1b) and provide oscillatory pressure pulses (Supplementary Figure S1c). The fluidic layer used channels with a rectangular cross section (250 μm wide), which allowed for the creation of partially closed sieve valve to form a packed bed against.

The protein purification was performed in the following four major steps: bead loading, protein adsorption, washing, and elution (Fig. 3b). We used three different workflows in the optimization of this process, which varied in the protein adsorption and washing steps. Workflow 1 used flow protein adsorption and flow washing. Workflow 2 used flow protein adsorption and oscillatory washing. Workflow 3 used oscillatory adsorption and washing. Step 1 (bead loading): Ni-NTA (nickel-nitrilotriacetic acid) magnetic beads (~3 μm in diameter) were flowed into the purification chamber and captured by the sieve valve or a magnet, for flow or oscillatory adsorption, respectively. Step 2 (protein adsorption): For flow adsorption (used in workflow 1 and 2), 10 µl of CFPS purification buffer, containing synthesized GFP, was flowed into the chamber through the packed bed of beads at a flow rate of 1 µl/min, allowing the His-tagged GFP to selectively bind to the Ni-NTA beads. For oscillatory adsorption (used in workflow 3), the 10 µl of CFPS purification buffer, containing produced GFP, was placed into the attached tubing where the protein solution could be passed through the magnetically immobilized beads multiple times (once every 30 s). Step 3 (washing): For flow washing (used in workflow 1), purification buffer was flowed at 5 µl/min through the packed bed of beads. For oscillatory washing (used in workflow 2 and 3), purification buffer is loaded into the attached tubing, where, by applying oscillating pressure pulses on the ends of the chamber, the beads would be moved back and forth in the chamber, inducing a shear stress on the bead surface and improving the washing rigor^26,28^. Step 4 (elution): 20 µL elution buffer was fed into the chamber through the packed bed of beads at a rate of 1 µl/min. During this step, purified GFP was released from the bead surface and removed from the chip for analysis.

We varied the conditions that potentially affected the result of protein purification. By systematically optimizing the amount of beads, binding buffer composition (e.g., adding 0.05% Tween-20), and workflows, we were finally able to achieve a product purity as high as 98.5% (Fig. 3c) while recovering 54.6% of the synthesized protein (Fig. 3d). Our on-chip protein purification system outperformed the off-chip system, conducted using the same beads following the manufacturer’s protocol, which only recovered 39.6% of proteins with a similar purity. It is also worth noting that the entire on-chip protein purification was finished in <40 min, where the off-chip protocol requires between 1 and 1.5 h. We found, through this set of optimizations, that the use of a surfactant (Tween-20) in the purification buffer had little effect on the recovery of the synthesized protein, but allowed for smoother and more reproducible operation of the microfluidic device. Without the addition of a surfactant in the system, the micromechanical valves occasionally became stuck in their position. Likewise, the use of oscillatory washing had little effect on yield but helped improve the consistency of the purification process. Finally, the use of the oscillatory adsorption drastically improved the overall yield of the purification module (50% recovery vs. 29% without oscillatory adsorption, on average). However, the result was also highly dependent on the amount of time used for the oscillatory adsorption step (50% for 10 min loading vs. 23% for 5 min).

**Fig. 4:**
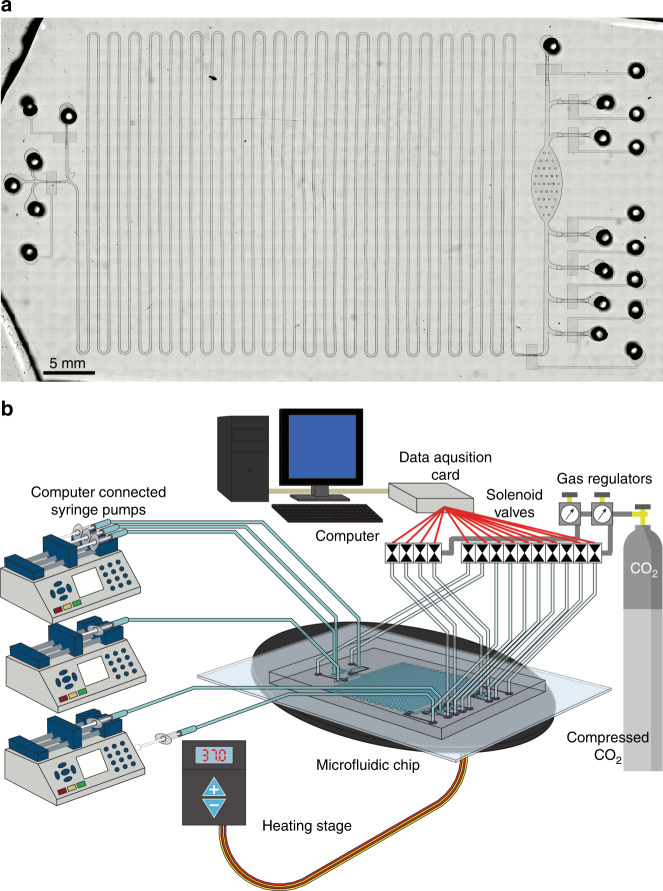
The integrated system for cell-free protein synthesis and purification. **a** Micrograph of integrated CFPS + P platform. **b** Overview of integrated CFPS + P platform setup. The system consists of computer-controlled solenoid valves and syringe pumps connected to a microfluidic device placed on a heating stage

**Fig. 5:**
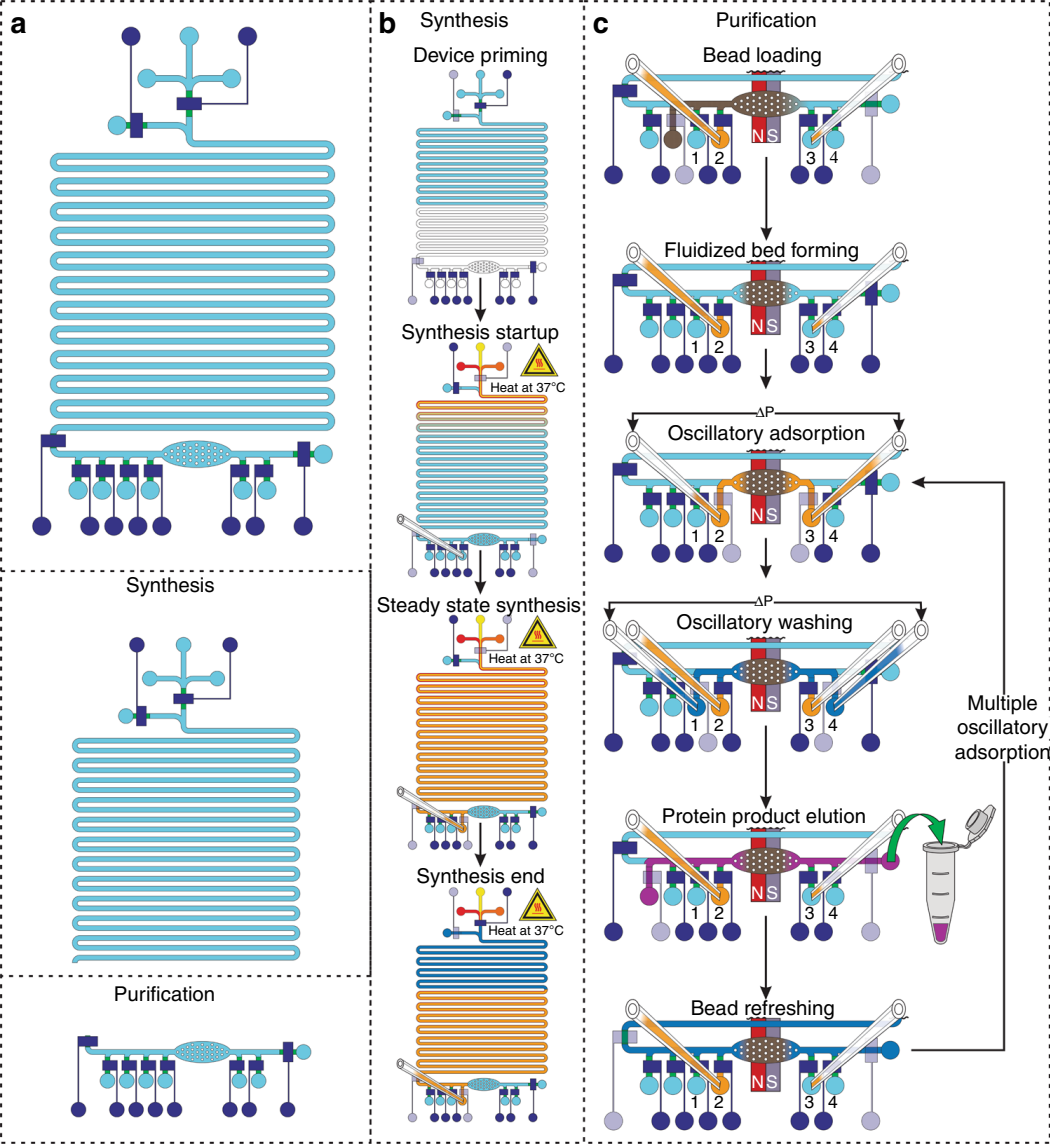
Overview and operation of the integrated cell-free protein synthesis and purification platform in 5 major steps: priming, protein synthesis, protein adsorption, washing, and elution, with an optional 6th step of bead refreshing. Dark blue denotes a closed valve, where transparency denotes an open valve. **a** Overview of integrated synthesis platform with identification of the synthesis and purification modules. **b** Synthesis operational workflow. Priming: Water is flowed into the system to prime the channel. Synthesis: The CFPS reagents are flowed into the individual inlets while being mixed and heated in the 13.2 μl reaction channel. The CFPS reaction mixture continuously flows into the attached tubings for storage. Once the desired amount of the CFPS reagents has been flowed into the system, purification buffer is flowed to force the remaining CFPS reaction mixture into tubing reservoir 2. **c** Purification operational workflow. Protein adsorption: After beads are flowed into the purification chamber and magnetically immobilized, the CFPS reaction mixture is loaded and the synthesized protein is adsorbed onto the beads during oscillatory adsorption. Washing: The protein-bead complexes are washed using oscillatory washing. Elution: The sythesized protein is eluted off of the bead surface. Bead refreshing: The beads are optionally refreshed for adsorption by flowing loading buffer through the bed of beads. Then the purification steps can be repeated, if desired

In phase 3, we developed an integrated CFPS + P microfluidic platform that integrates CFPS with protein purification (Figs. 4a and 5a). Our device was designed by combining the basic working principles of our preliminary studies, with some alterations made for integration and automation. In this system, we combined a continuous-flow reactor and a batch purification device. These two processes are not intrinsically compatible with each other and such integration has not been previously demonstrated. To accomplish this integration, we interfaced the two processes using a tubing reservoir that stored the continuously produced protein on chip before it was purified. The device was operated using multiple solenoid valves and computer-controlled syringe pumps (Fig. 4b). All of the ancillary apparatus required to operate the microfluidic system can potentially be confined to the size of a briefcase, which makes it a highly portable system for therapeutic protein production.

The operation of the integrated CFPS + P chip is summarized in Fig. 5 and described in great detail in the methods section. The operation consisted of 5 major steps: priming, protein synthesis, protein adsorption, washing, and elution, with an optional 6th step of bead refreshing. The synthesis steps are described in Fig. 5b while purification is in Fig. 5c. Step 1 (priming): The system was evacuated of air by filling the channels with water. This ensured that no air bubbles caused blockages in the system. Step 2 (protein synthesis): The protein was synthesized in the serpentine synthesis channel as in the individual synthesis module. The synthesized protein was stored in tubing reservoir 2, which was upstream of the purification module, until the total synthesis volume of 25 μl has been reached. The protein was synthesized at a rate of 13.2 μl of synthesized protein per 1.5 h, with the whole production taking ~4.5 h accounting for startup time (1.5 h startup time). The startup time was the amount of time required between the synthesis step starting and the synthesized protein reaching the end of the synthesis channel after displacing water in the channel. After the desired amount of protein was synthesized, the synthesis module was closed off from the purification module by closing the micromechanical valve between the 2 modules.

In the isolated protein purification module, we conducted protein purification using a magnetically immobilized bed instead of the sieve valve mechanically packed bed used in the previous development to avoid high pressure inherent in the packed bed system. Step 3 (protein adsorption): After loading and magnetically immobilizing our Ni-NTA magnetic beads in the purification chamber, the entire volume of synthesized protein mixture stored in tubing reservoir 2 was flowed through the packed bed of beads in 30 s pulses (back and forth between reservoirs 2 and 3) under a low pressure of ~2 psi, exposing the entire solution volume to the packed bed of beads. This process was conducted for 10 min, in which the entire protein solution passed through the magnetically immobilized bed of Ni-NTA beads 20 times. Step 4 (washing): The protein-bound beads were washed using an oscillatory washing step similar to the one used in Fig. 3b. The oscillatory washing tubings were preloaded with 30 μl of purification buffer each and then closed off from the system using on-chip micromechanical valves while not in use. The purification buffer was oscillated under a pressure of ~5 psi, in 0.5 s pulses between reservoirs 1 and 4. The oscillatory washing was performed for 5 min. Step 5 (elution): The purified protein was eluted from the bead surface by flowing elution buffer through the magnetically packed bed of beads at a flow rate of 1 μl/min for 20 min. The elution buffer, containing a high concentration (300 mM) of imidazole, is well established to dissociate the protein-bead interactions and return the target protein to the mobile phase in active form^29^. Step 6 (bead refreshing): Following the elution, the Ni-NTA beads could be refreshed by flowing purification buffer through the magnetically immobilized bed at a rate of 5 μl/min for 5 min. The refreshed beads could then be used to perform another round of adsorption, washing, and elution in order to improve the protein recovery. Each pass of the purification module took ~40 min to perform.

**Fig. 6:**
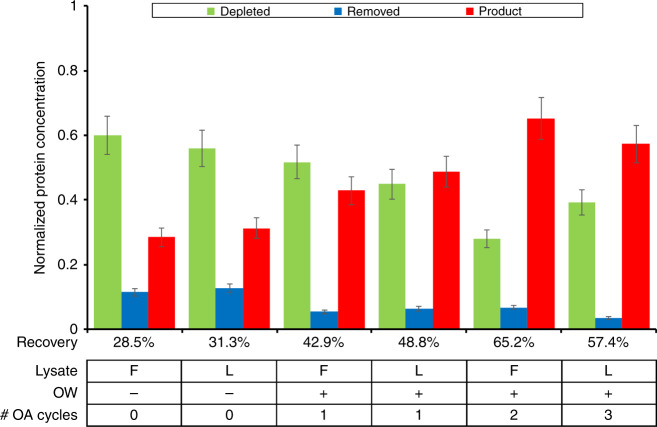
GFP synthesis and purification using integrated CFPS + P microfluidic device. In all experiments, the purification and elution buffers contained 0.5% Tween-20 and 5 μl Ni-NTA beads were used. The optimization of the integrated CFPS + P device was conducted by examining three conditions. (1) Use of fresh (F) or lyophilized (L) lysate; (2) Use the 5-min oscillatory washing (OW). (3) Number of cycles for oscillatory adsorption (OA). Depleted: CFPS reaction mix after bead absorption; removed: removed contaminates in the purification buffer; product: purified GFP in the elution buffer

We first optimized the workflow of the CFPS + P platform using GFP (Fig. 6). We tested a number of different conditions and their effects on the recovery of purified protein. Using fresh or lyophilized lysate seemed to have little effect on the overall recovery rate of purified protein. The use of oscillatory washing and adsorption had a much larger effect on the recovery of synthesized protein, comparatively. Oscillatory adsorption improved the recovery by an average of 15%. The introduction of multiple oscillatory adsorptions reduced the amount of uncaptured protein by as much as half, but additional cycles, while increasing the amount of protein captured, had diminishing returns. We suspect this is a result of the surface of the Ni-NTA beads being fouled to a greater extent through each cycle. As expected, the use of the integrated platform had no noticeable effect on the purity of the protein compared to the individual purification module, producing GFP with a purity of 98%.

**Fig. 7:**
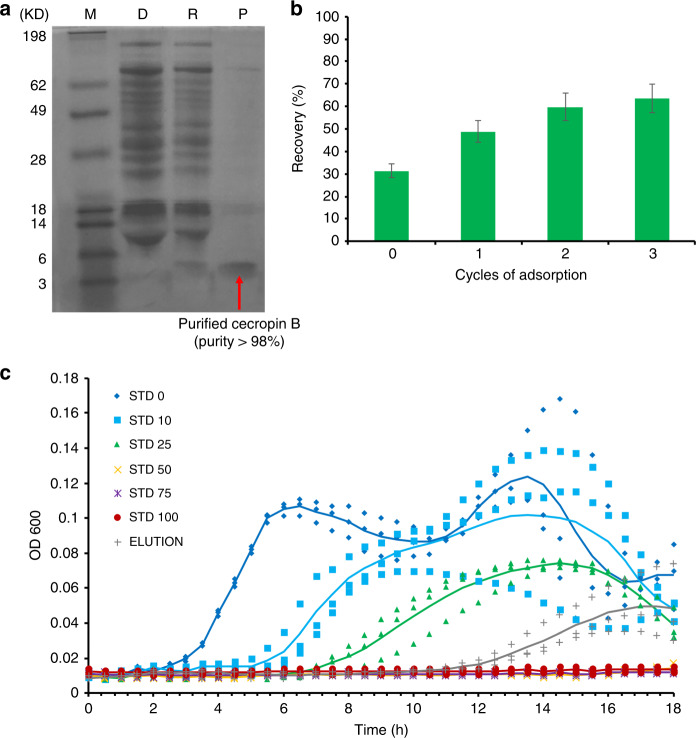
Cecropin B synthesis and purification using integrated CFPS + P microfluidic device. In these experiments, the purification and elution buffers contained 0.5% Tween-20, 5 μl Ni-NTA beads were used, as well as oscillatory washing and adsorption. **a** Tricine-PAGE of the cecropin B synthesized and purified by the integrated chip. M (marker); D (depleted): CFPS reaction mix after bead absorption; R (removed): removed contaminates in the purification buffer; P (product): purified AMP in the elution buffer. **b** Optimization of cecropin B recovery using multiple adsorption cycles. **c** Growth inhibition assays of *E. coli* conducted in triplicate, using the protein product (elution) after three adsorption/elution cycles by our device in comparison to cecropin B standards of different concentrations (0–100 ng/μl, denoted as STD 0-100). OD600 was measured every 30 min for 18 h. Elution with a concentration of 27 ng/μl in 60 μl solution was used. Trend lines that represent the average of three trials are added to guide the eye

After optimizing the workflow of the CFPS + P microfluidic platform using GFP, we applied the optimized conditions to synthesize one antimicrobial peptide, cecropin B^30^, using the integrated CFPS + P microfluidic system (Fig. 7). Antimicrobial peptides are a diverse class of peptides that serve as defense molecules against infection by interfering with protective cell layers and intracellular components^31,32^. Given their wide target range (bacteria, viruses, fungi, and cancer) and compact molecular structure (<10 kDa), they make an ideal therapeutic protein candidate for synthesis. DNA templates encoding cecropin B were used instead of GFP templates in this experiment. We used an elution volume of 20 μl, where one device was capable of performing multiple adsorption/elution cycles. Following subsequent expression, purification, electrophoresis, and staining, we were able to confirm the successful production and purification of cecropin B. Using the cecropin B standards (Sigma), semi-quantitative analysis was applied to the purified cecropin B. The recovery of soluble peptides in the first elution was 63 ng/μl with a purity of 92% for cecropin B (Fig. 7a). In addition, we were able to demonstrate the ability to increase our protein recovery by increasing the number of adsorption cycles used (Fig. 7b). As we saw with GFP adsorption, we did notice diminishing returns with each subsequent adsorption cycle. Finally, cecropin B bioactivity was tested and exhibited a growth inhibition effect on *E. coli* TOP10 (Fig. 7c). The successful inhibition of *E. coli*. demonstrated that the cecropin B produced by our microfluidic system was still active and could effectively suppress bacterial growth.

## Discussion

We have effectively demonstrated the manufacturing and testing of a microfluidic system capable of synthesizing and purifying therapeutic proteins using either fresh or lyophilized reagents, with a high purity and at therapeutically relevant concentrations. In addition, we showed the ability of our produced therapeutic protein to effectively retard the growth of *E. coli* in culture, demonstrating its effectiveness as a therapeutic agent. Our system is low-cost, highly integrated, and ideal for low-resource settings.

In future work, the microfluidic device and its ancillary control system will benefit from continued improvement in automation and integration. In addition to optimization of the on-chip design and processes, the control system can be integrated into a portable and affordable platform that facilitates running the specific microfluidic processes involved. Similar integrated systems can also be created for other proteins that would benefit from point-of-care production.

## Materials and methods

### In-house cell-free extract preparation

*E. coli* BL21 was grown in 400 ml of LB containing chloramphenicol (34 mg/ml) and 0.1 mM IPTG at 37 °C at 250 rpm. Cells were harvested in mid-exponential growth phase (OD600 0.6), and cell pellets were washed three times with ice cold buffer A (10 mM Tris-Acetate pH 8.2, 14 mM magnesium acetate, 60 mM potassium glutamate, and 1 mM DTT). Cell extract was prepared as described in a previous publication^33^. In summary, the cell pellets were thawed and resuspended in 1.3 ml of buffer A per gram of wet cells and homogenized by French press once. The lysate was centrifuged at 30,000 g for 30 min at 4 °C. The supernatant was collected and incubated at 37 °C at 300 rpm for 1 h. The supernatant was centrifuged again at 30,000×*g* for 30 min at 4 °C, flash frozen with liquid nitrogen, and stored at −80 °C until use. Using a previously published cell-free reaction protocol^25^, the final reaction mixture was composed of equal parts in-house lysate, DNA template (plasmid) (12 μg/ml), and CFPS reaction buffer (1.2 mM adenosine triphosphate (ATP); 0.85 mM each of GTP, UTP, and CTP; 34 μg/ml folinic acid; 170.6 μg/ml of *E. coli* tRNA mixture; 2 mM each of 20 unlabeled amino acids; 0.33 mM nicotinamide adenine dinucleotide (NAD); 0.26 mM coenzyme-A (CoA), magnesium acetate 3.3 mM, magnesium glutamate 20 mM, ammonium glutamate 10 mM, potassium acetate 14.4 mM, potassium glutamate 175 mM, phosphoenolpyruvate 33 mM, spermidine 1.5 mM, putrescine 1 mM, sodium oxalate 2.7 mM, Tris (pH 8.2) 2.4 mM)

### Microfluidic device fabrication

Three different microfluidic systems were demonstrated and fabricated in this work. The synthesis platform consisted of a 200 μm wide channel that is 50 μm deep with a total volume of 13.2 μl (Fig. 2a). The purification platform consisted of a single oval shaped chamber with a microfluidic sieve valve (Fig. 3a). The integrated platform combined the synthesis platform with the same dimensions and the oval purification chamber with additional inlets for oscillating washing and adsorption as well as reagent loading (Fig. 5a). The photomasks were designed using LayoutEditor and plotted by Fineline Imaging onto Mylar transparencies with a resolution of 10 K DPI. The Mylar photomasks were used to produce silicon masters using photolithography. The channels and purification chambers (in the fluidic layer) were all fabricated using SU-8 2025 (MicroChem) according to manufacturer instructions for a depth of 50 μm and subsequently hard baked for 30 min at 250 °C. The purification and integrated devices were fabricated using multilayer soft lithography^18,26–28,34^. In these cases, after the SU-8 layer was patterned in the fluidic layer master, an AZ layer was patterned using AZ 9260 (EMD Performance Materials) at a depth of 25 µm. After development, the fluidic master was heated at 130 °C for 1 min in order to create rounded AZ channels. The control layer master was fabricated in SU-8 with a thickness of 30 µm. The silicon masters were then used as molds for polydimethylsiloxane (PDMS RTV 615, Momentive Performance Materials) which was mixed in a ratio of 5:1 prepolymer to crosslinking agent and poured in bulk onto the fluidic layer and 20:1 for the control layer membrane. The mixed PDMS was vacuumed for 1 h before the control layer membrane was spun at a speed of 500 RPM for 10 s followed by 1750 RPM for 30 s. The fluidic and control layers were baked in a 75 °C oven for 12 min before the bulk fluidic layer was removed from the mold and holes were punched for the inlets and outlets (Harris Uni-Core). The fluidic layer was aligned and thermally bound to the thin control layer in the 75 °C oven for 1 h. The multilayer device was removed from the mold and holes were punched for the control layer before the device was bound to a glass slide (pre-cleaned using dish soap) using an oxygen plasma treatment (Harrick Plasma) with the control layer sandwiched between the fluidic layer and the glass slide. The devices were then cured in the 75 °C oven for another 30 min before being used. Our devices were designed for single use.

### Synthesis device operation

The synthesis device was set up connected to a syringe pump (Chemyx) and placed on a stage heater (WP-10, Warner Instruments) (Supplementary Figure S1a). The synthesis device was operated by flowing the DNA template (plasmid), CFPS buffer (energy solution), and lysate into the microfluidic device while heating at 37 °C, as shown in Fig. 2b. We utilized a continuous-flow system, where we flowed the reagents at a total flow rate of 0.15 μl/min (0.05 μl/min per buffer), this which resulted in an effective 1.5 h incubation time and allowed for a higher volume of protein synthesis than could be done with a batch system.

### Purification device operation

The device setup consisted of the microfluidic chip connected to two sets of different pressure controlled solenoid valves (ASCO) and a syringe pump (Chemyx), all controlled by a custom LabVIEW program (Supplementary Figure S1b, c). The individual purification device was based on adsorption/washing mechanisms demonstrated previously by our group^26^. The micromechanical valves were operated at a pressure of 30 psi. The oscillation steps were operated by applying alternating 2 and 5 psi pressure pulses at the opposite ends of the purification chamber for oscillatory adsorption and washing, respectively. The purification device was operated in three different workflows (Fig. 2b). Workflow 1: Flow adsorption and washing. Workflow 2: Flow adsorption and oscillatory washing. Workflow 3: Oscillatory adsorption and washing. The purification device was primed with purification buffer (10 mM imidazole, 300 mM NaCl, 50 mM sodium phosphate (pH 8.0), 0.05% Tween-20) and evacuated of air bubbles. Ni-NTA magnetic beads (G-Biosciences) were loaded into the purification chamber. For flow adsorption, the protein was flowed into the chamber at 1 μl/min through the bed of Ni-NTA magnetic beads, mechanically packed against the sieve valve. For oscillatory adsorption, the protein solution was loaded into the low pressure tubing (IDEX) and loaded for 5 or 10 min exposing all of the protein solution to the beads 10 or 20 times, respectively, which are magnetically immobilized. After the adsorption step, the bead complexes were washed with purification buffer. For flow washing, purification buffer was flowed through the packed bed of Ni-NTA beads at a flow rate of 5 μl/min for 5 min. For oscillatory washing, purification buffer was loaded into the tubings at either end of the purification chamber, where alternating 0.5 s pressure pulses wash the bead complexes for 5 min over 300 oscillatory cycles. After washing, the protein was eluted into 20 μl elution buffer (300 mM imidazole, 300 mM NaCl, 50 mM sodium phosphate (pH 8.0), 0.05% Tween-20) by flowing at 1 μl/min through a packed bed of beads. The eluted protein could then be analyzed off-chip for purity and concentration.

### Integrated device operation

The microfluidic system consisted of a PDMS microfluidic device, stage heater (WP-10, Warner Instruments), set of syringe pumps (Chemyx), and solenoid valves (ASCO) controlled by a custom LabVIEW program placed on a microscope stage (Olympus) (Fig. 4b). The solenoid valves were controlled by 2 different regulators, allowing us to operate our micromechanical valves at a constant pressure of 30 psi and our oscillating pulses at 2 and 5 psi, for loading and washing respectively. The operation of the system is summarized in Fig. 5b for synthesis and Fig. 5c for purification. The control layer structures were filled with water and cleared of air before starting operation. The fluidic layer of the device was also purged of air with water. The tubing system could be connected or closed off from the system using on-chip micromechanical valves. 1 μl of water was flowed into each tubing after they were connected to the device and kept in there by closing the micromechanical valves before washing. The device was moved onto the 37 °C stage heater and CFPS buffer (energy solution), DNA template (plasmid), and lysate were flowed at equal volumetric flow rates with a combined flow of 0.15 μl/min (0.05 μl/min per buffer). First, the flow was directed to the outlet, until all water was displaced from the synthesis channel, then the flow was directed to the reservoir, collecting the synthesized protein in reservoir 2. This flow rate allowed for a 1.5 h incubation at 37 °C in the synthesis channel before the protein was flowed into the oscillatory adsorption tubing reservoir. The flow changed to purification buffer after loading the desired amount of synthesis reagents, in order to conserve reagents. After synthesis of the desired amount of protein product, purification buffer was loaded into the oscillatory washing tubing, and 5 μl of Ni-NTA magnetic beads, which were first washed twice with 100 μl of purification buffer, were loaded into the purification chamber. The beads were magnetically immobilized and the protein product was adsorbed through oscillation, between reservoirs 2 and 3, allowing for the full product to contact the beads for 10 min total. After adsorbing the protein onto the beads, they were washed with purification buffer oscillated using 0.5 s oscillating pulses, between reservoirs 1 and 4, at 5 psi for a total of 300 oscillations in 5 min. The beads were then collected in the purification chamber and formed into a magnetically immobilized bed by placing a magnet under the chamber and eluted for 20 min at a flow rate of 1 μl/min. The beads were then refreshed by flowing 25 μl of purification buffer at 5 μl/min for 5 min through the magnetically packed bed. With the refreshed beads, we can repeat the steps of protein adsorption, washing, and elution as previously described in order to improve recovery.

### GFP plasmid construction

The eGFP was amplified by PCR with the forward primer (CCGCTCGAGCGATGTCTAAAGGTGAAG) and reverse primer (CGCGGATCCTTATTTGTACAATTCATCCATACC). The amplified PCR product and the plasmid pIVEX2.4c were digested by *Xho*I and *Bam*HI restriction enzymes (New England Biolabs). The larger fragment of pIVEX2.4c was recycled by DNA gel purification kit and the digested PCR fragment purified by PCR purification kit (Qiagen). These two fragments were mixed together and ligated by T4 DNA ligase (New England Biolabs). After transformation, the correct transformants were identified by restriction enzyme digestion.

### Cecropin B plasmid construction

The cecropin B was synthesized by IDT gBlock with the sequence reported previously^7^. Plasmid pIVEX2.4c was used as the backbone to insert the whole expression cassette. The plasmid pIVEX2.4c and the synthesized expression cassette were digested by *Hin*dIII and *Eco*RI restriction enzymes (New England Biolabs). The larger fragment of pIVEX2.4c was recycled by DNA gel purification kit and the digested cecropin B fragment was purified by PCR purification kit (Qiagen). These two fragments were mixed together and ligated by T4 DNA ligase (New England Biolabs). After transformation, the correct transformants were identified by restriction enzyme digestion.

### Preparation of lyophilized lysate

Lyophilization took place in 0.5 ml volumes in accordance with previous work^35^. Samples were loaded into 70 ml cylindrical glass vials for shell freezing in a −80 °C freezer (Thermo) and incubated for a minimum of 5 min. Vials were transferred to the freeze dryer (Flexi-dry MP, FTS Systems) for 20 min periods. The operating conditions of the freeze dryer were −60 °C and <120 mTorr, with a 19–20 °C ambient temperature. At the end of each 20 min drying period, vials were placed in the −80 °C freezer for 10 min and subsequently placed onto the freeze dryer. Lyophilization continued in this manner until at least 95% of the estimated water mass was lost (typically three cycles), at which time the vials were chilled in the freezer for 10 min, then placed back on the freeze dryer for an additional 60 min to provide for removal of the more tightly interacting water molecules. The lyophilized product was stored in sealed microcentrifuge tubes.

### SDS-PAGE and western blotting

The expressed protein was analyzed by SDS-PAGE electrophoresis and Western blotting. For Western blotting analysis, the target gel was transferred onto a 0.45-μm nitrocellulose (NC) membrane (Pall), then the membrane was incubated with primary mouse anti-His antibody (Qiagen) and HRP-conjugated secondary antibody of goat anti-mouse IgG subsequently. Finally, the membrane was visualized by DAB. The quantity of target protein was calculated by Quantity One (Bio-Rad). Cecropin B samples were boiled for 3 min in 2× Tricine sample buffer (Bio-Rad; 161-0739), loaded in 16.5% polyacrylamide Tris/Tricine precast gel, and run at 200 mA for 4.5 h. For Coomassie staining, gels were fixed for 1 h in 12% trichloroacetic acid and 1 h in 40% ethanol and 10% acetic acid, followed by 14 h staining in QC Colloidal Coomassie, and 2 h de-staining in water.

### Growth inhibition assays for cecropin B

*E. coli* Top 10 were grown overnight at 37 °C in LB, diluted 1:100, grown to OD600 0.5, diluted to 10^4^ cells/ml in LB containing 0.005% Antifoam 204 to eliminate micro-bubbles, and distributed into wells of a 96-well plate containing 10 μl of the CFPS + P cecropin B, standards, or water. All wells were overlaid with 20 μl mineral oil to prevent evaporation and condensation. OD600 was recorded every 30 min for 18 h at 37 °C using a plate-reader.

## Supplementary information

Supplementary Information
