# Supplementary material for: On-chip manufacturing of synthetic proteins for point-of-care therapeutics
Source: Microsyst Nanoeng. 2019 Mar 25;5:13. doi: 10.1038/s41378-019-0051-8 (PMC6431678; doi:10.1038/s41378-019-0051-8)
Supplement: Supplementary file 1 — Supplementary Information [file 41378_2019_51_MOESM1_ESM.pdf]

Supplementary Information

On-chip Manufacturing of Synthetic Proteins for Point-of-care Therapeutics

Travis W. Murphy,[†,a] Jiayuan Sheng,[†,b] Lynette B. Naler,[a] Xueyang Feng,[b] Chang Lu*[,a]

[a] Department of Chemical Engineering, Virginia Tech, Blacksburg, VA 24061

[b] Department of Biological Systems Engineering, Virginia Tech, Blacksburg, VA 24061

[†] These two authors contributed equally

* To whom correspondence should be addressed. E-mail: changlu@vt.edu

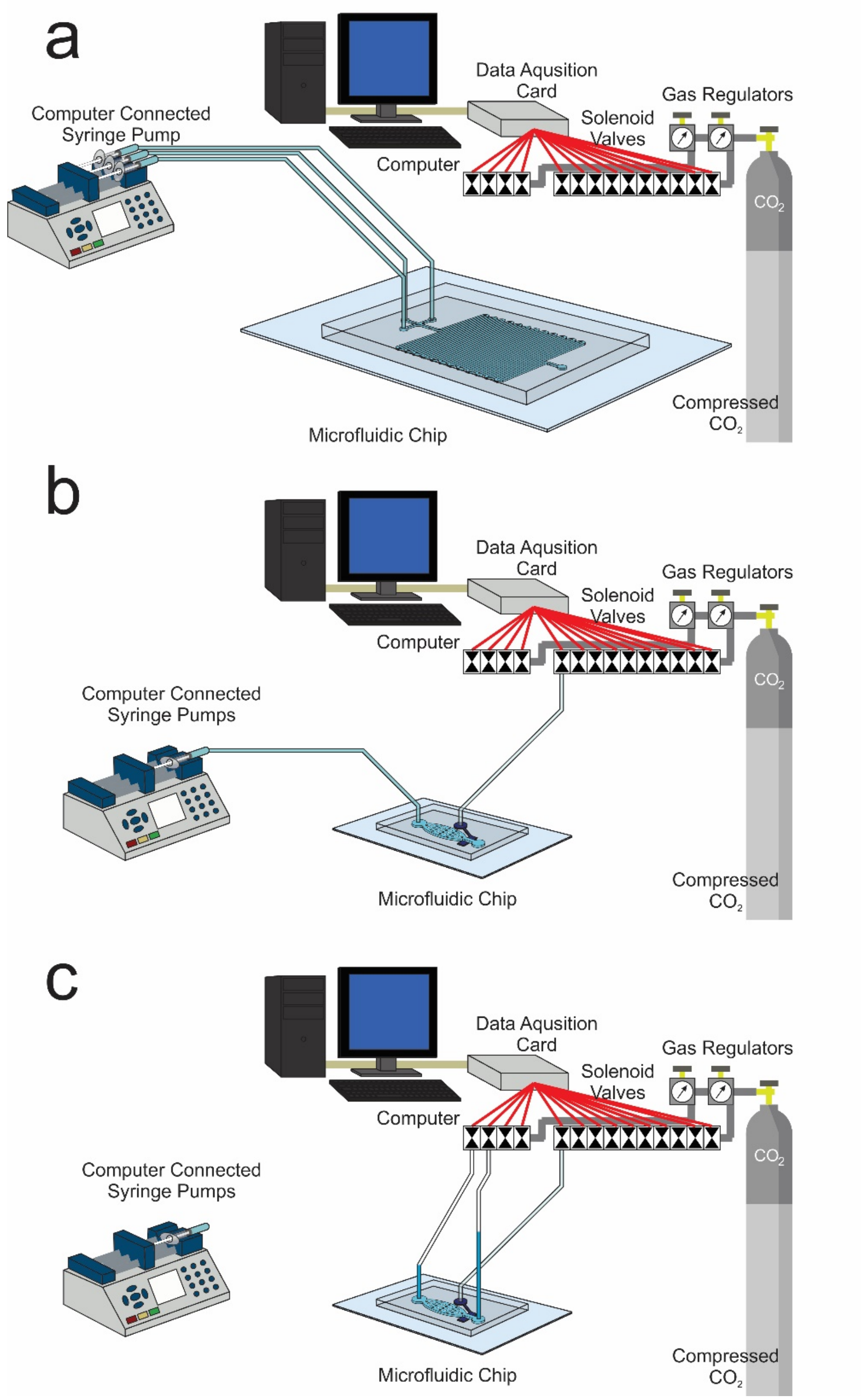

Figure S1 Overview of protein synthesis and purification device setups. (a) Setup of the synthesis device utilizing only one syringe pump. (b) Setup of the purification device for the flow adsorption of chromatin. (c) Setup of the purification device with computer-controlled solenoid valves for oscillatory adsorption or washing.

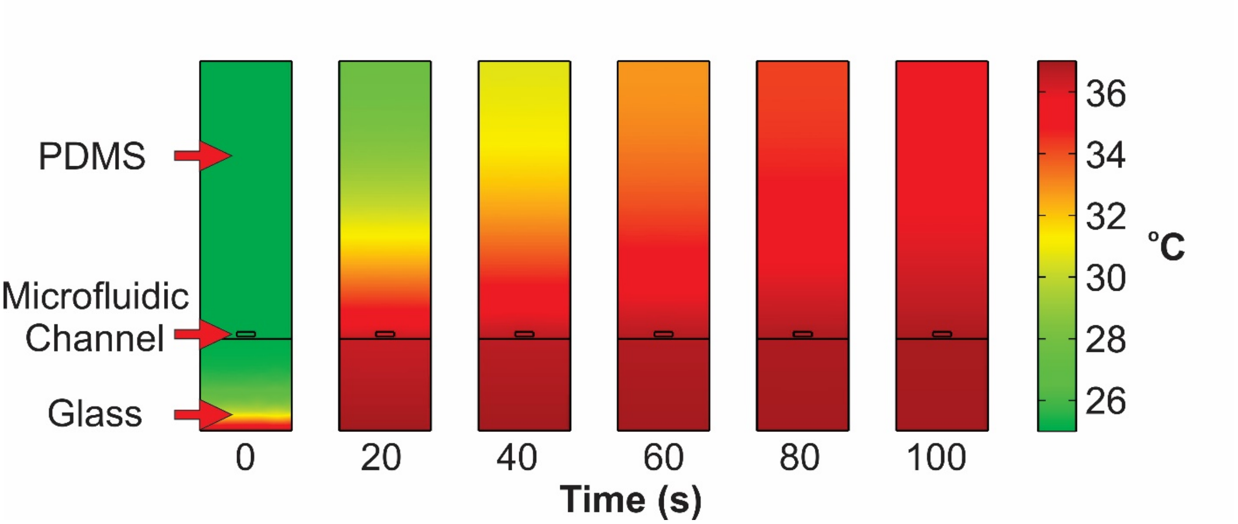

Figure S2 COMSOL model of microfluidic channel heating over time using a constant 37 °C boundary condition against the glass slide.
